# Hesperidin Mitigates Cyclophosphamide-Induced Testicular Dysfunction via Altering the Hypothalamic Pituitary Gonadal Axis and Testicular Steroidogenesis, Inflammation, and Apoptosis in Male Rats

**DOI:** 10.3390/ph16020301

**Published:** 2023-02-15

**Authors:** Tarek Khamis, Abdelmonem Awad Hegazy, Samaa Salah Abd El-Fatah, Eman Ramadan Abdelfattah, Marwa Mohamed Mahmoud Abdelfattah, Liana Mihaela Fericean, Ahmed Hamed Arisha

**Affiliations:** 1 Department of Pharmacology and Laboratory of Biotechnology, Faculty of Veterinary Medicine, Zagazig University, Zagazig 44519, Egypt; 2 Anatomy and Embryology, Faculty of Dentistry, Zarqa University, Zarqa 13110, Jordan; 3 Human Anatomy & Embryology Department, Faculty of Medicine, Zagazig University, Zagazig 44519, Egypt; 4 Biology Department, Faculty of Agriculture, University of Life Sciences “King Michael I of Romania” from Timisoara, Aradului St. 119, 300645 Timisoara, Romania; 5 Department of Animal Physiology and Biochemistry, Faculty of Veterinary Medicine, Badr University in Cairo, Badr City 11829, Egypt; 6 Department of Physiology, Laboratory of Biotechnology, Faculty of Veterinary Medicine, Zagazig University, Zagazig 44519, Egypt

**Keywords:** cyclophosphamide, hesperidin, apoptosis, P53, iNOS, oxidative stress

## Abstract

Cyclophosphamide (CP) is a cytotoxic, cell cycle, non-specific, and antiproliferative drug. This study aimed to address the toxic effects of CP on male fertility and the possible ameliorative role of hesperidin (HSP). Thirty-two adult albino rats were randomly divided into four groups, namely, the negative control, HSP, CP-treated, and CP+HSP-treated groups. The CP-treated rats showed a significant reduction in the levels of serum LH, FSH, testosterone, prolactin, testicular glutathione peroxidase (GPx), and total antioxidant capacity (TAC) with an elevation in levels of malondialdehyde (MDA), and p53, and iNOS immune expression, compared to the control group. A significant downregulation in hypothalamic KISS-1, KISS-1r, and GnRH, hypophyseal GnRHr, and testicular mRNA expression of steroidogenesis enzymes, PGC-1α, PPAR-1, IL10, and GLP-1, as well as a significant upregulation in testicular mRNA of P53 and IL1β mRNA expression, were detected in the CP-treated group in comparison to that in the control group. The administration of HSP in CP-treated rats significantly improved the levels of serum LH, FSH, testosterone, prolactin, testicular GPx, and TAC, with a reduction in levels of MDA, and p53, and iNOS immune expression compared to the CP-treated group. A significant upregulation in hypophyseal GnRHr, and testicular mRNA expression of CYP19A1 enzymes, PPAR-1, IL10, and GLP-1, as well as a significant downregulation in testicular mRNA of P53 and IL1β mRNA expression, were detected in the CP+HSP-treated group in comparison to that in the CP-treated group. In conclusion, HSP could be a potential auxiliary agent for protection from the development of male infertility.

## 1. Introduction

The incidence of cancer has increased significantly throughout the world in recent years [1]. Formerly, surgery used to be the sole option for treating patients with solid tumors, which had a high fatality rate. Chemotherapy has improved such patient survival rates over the past 40 years [2]. Various chemotherapeutic agents, including cyclophosphamide (CP), are widely used as a part of their treatment regimen [3]. Unfortunately, it is non-selective for diseased cells [4,5]. CP has two active metabolites, namely, phosphoramide mustard and acrolein. While phosphoramide mustard has been associated with the antineoplastic and immunosuppressive actions of CP, acrolein is responsible for the toxic side effects of CP that include apoptosis and necrosis in normal tissue [6]. CP administration has been linked to significant side effects, such as hemorrhagic cystitis, gonadotoxicity, nephrotoxicity, and cardiotoxicity, limiting its therapeutic use. Moreover, female patients who are treated with CP are susceptible to early menopause. The risk of irreversible infertility increases significantly in both male and female patients when exposed to a cumulative medical dose [7,8]. CP has been reported to impact male fertility both centrally and peripherally via downregulating the hypothalamic–pituitary–gonadal (HPG) axis through affecting the hypothalamic Kiss1 mRNA expression and gonadotropin secretion, testicular steroidogenesis, testosterone synthesis, and eventually spermatogenesis [9,10]. Furthermore, Cp has been linked to direct testicular oxidative stress and DNA damage leading to testicular degeneration [11,12].

Flavonoids, found in many medicinal plants, fruit, and vegetables, can be useful in treating a variety of diseases. Flavonoids possess various pharmacological properties, including vasodilation, anti-allergic, immunostimulant, and antiviral effects [13,14]. They are reported to be effective as an antioxidant, anticarcinogen, antiproliferative, and in combating multidrug resistance, as well as in preventing chemotherapy-associated injury [15]. Hesperidin (HSP) is a bioflavonoid that is found mainly in citrus fruit such as oranges and lemons, as well as plant-derived liquids such as tea and olive oil. It has a wide range of pharmacological effects, including antioxidant, anti-inflammatory, anticarcinogenic, antiviral, antibacterial, antifungal, antiulcer, analgesic, and anticancer properties [16]. The main objective of the current work was to examine the potential therapeutic effect of HSP in preventing the progression of male infertility in rats treated with CP and its effects on the various molecular processes involved in male reproduction.

## 2. Results

### 2.1. Effect on the Final Body, Testicular Weights and Serum Hormone Levels

**Figure 1:**
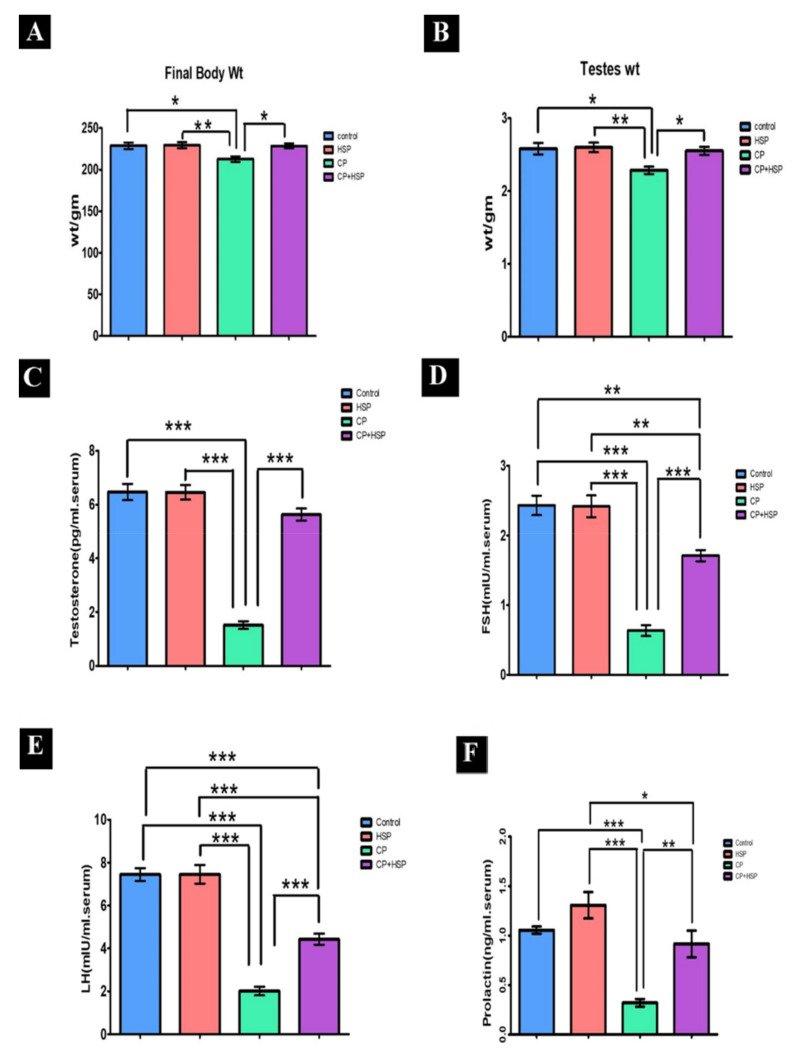
Effect on the final body, testicular weights and serum hormones (**A**–**F**). (**A**) Body weight (g), and (**B**) weight (g), (**C**) serum testosterone level (pg/mL), (**D**) serum FSH (mIU/mL) level, (**E**) serum LH (mIU/mL) level, and (**F**). serum prolactin (ng/mL) level. Data are expressed as means ± SEM. N = 8. *, **, *** indicate significant difference (*p* < 0.05).

The final body and testicular weights of the CP-treated group were significantly lower (*p* < 0.05) compared to the control group in Figure 1A,B. The CP+HSP-treated group showed a significant increase in final body and testicular weights compared to the CP-treated group in Figure 1A,B.

The serum levels of testosterone, FSH, LH, and prolactin showed a significant decrease in the CP-treated group in comparison to the control group (*p* < 0.05) in Figure 1C–F. The serum levels of testosterone, FSH, LH, and prolactin in the CP+HSP-treated group showed a significant increase compared to the CP-treated group, shown in Figure 1C–F.

### 2.2. Effect on Testicular Lipid Peroxidation and Oxidative Stress Markers

**Figure 2:**
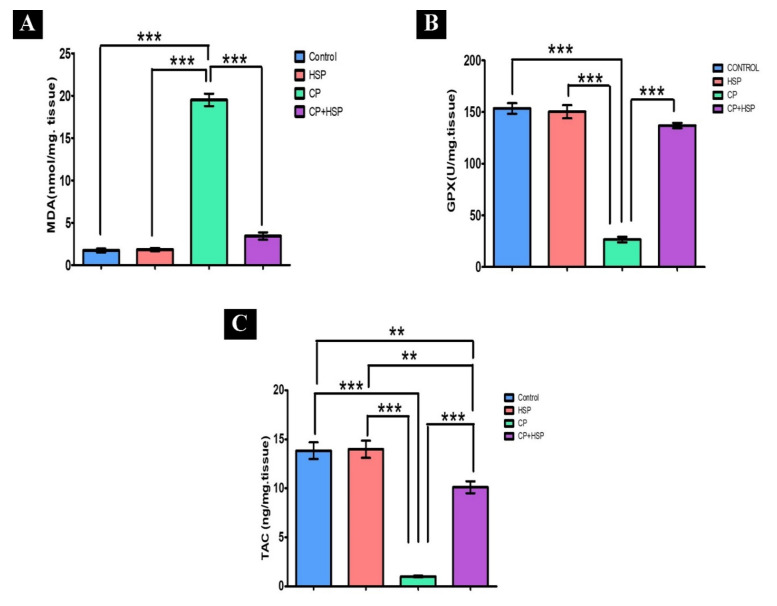
Effect of hesperidin administration in cyclophosphamide-induced testicular impairment in male rats on testicular lipid peroxidation and oxidative stress markers (**A**–**C**). (**A**) Testicular MDA level (nmol/mg. tissue), (**B**) testicular GPx level (U/mg. tissue), and (**C**) testicular TAC level (ng/mg. tissue). Data are expressed as means ± SEM. N = 8. **, *** indicate significant difference (*p* < 0.05).

CP-induced oxidative stress was indicated, with a significant decrease in GPx and TAC levels in the CP-treated group in comparison to that in the control group (*p* < 0.05), shown in Figure 2B,C. A significant increase in the MDA level, the lipid peroxidation marker, in the CP-treated group in comparison to that in the control group (*p* < 0.05) is also shown in Figure 2A. The CP+HSP-treated rats showed a significant decrease in testicular MDA levels, as well as a significant increase in testicular GPx and TAC levels, in comparison to those in the CP-treated group, shown in Figure 2A–C.

### 2.3. Effect on Testicular Histopathology and Morphology

**Figure 3:**
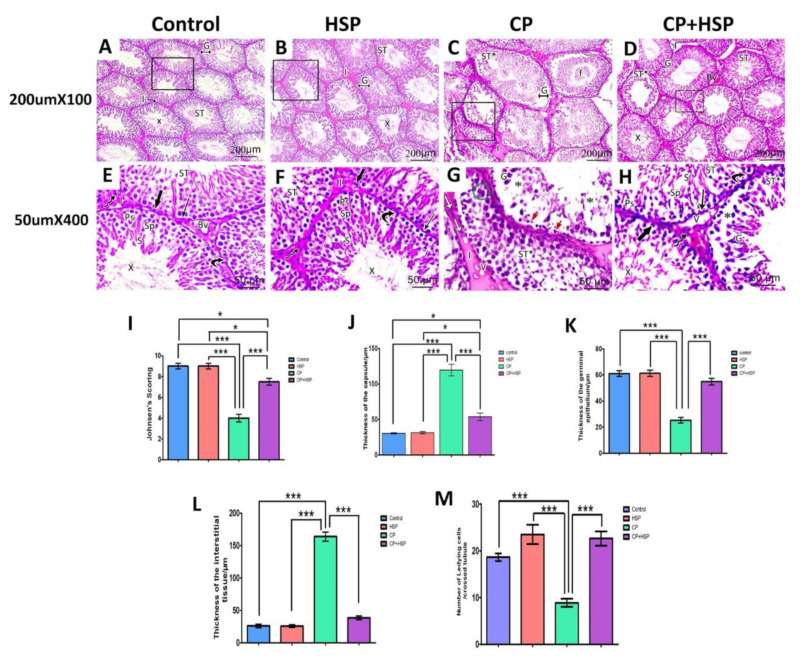
Effect of hesperidin administration in cyclophosphamide-induced testicular impairment in male rats on testicular histopathological and morphometric analysis (**A**–**M**). (**A**) Seminiferous tubules (ST) lined with stratified germinal epithelium (G). Aggregation of sperm is seen in their lumina (X). Narrow interstitial spaces (I), H&E X100. (**B**) Seminiferous tubules are closely packed (ST) and lined by stratified germinal epithelium (G). Clumps of sperm are seen in their lumina (X). A narrow interstitium is seen in between the tubules and contains clusters of cells (I), H&E X100. (**C**) Distorted seminiferous tubule (ST*) with irregular outlines and atrophied layers of germinal epithelium (G) with exfoliation of some germ cells towards the lumen (f). The interstitial tissues are wide and edematous (I), H&E X100. (**D**) Well-organized seminiferous tubules (ST) with a normal regular outline of germinal epithelium (G) and aggregation of sperm in their lumina (X). Other tubules appear affected with detached germinal epithelium and empty lumen (ST*). Relatively narrow interstitial spaces (I) between tubules and congested blood vessels (Bv*) are seen, H&E X 100. (**E**) The higher magnification of the figure (**A**) shows seminiferous tubules lined with; spermatogonia (thick arrow), primary spermatocytes (Ps), secondary spermatocytes (Sp), spermatids (S), and sperms (X) are seen. Sertoli cells (curved arrow) are resting on the basement membrane. The seminiferous tubule is ensheathed by a single layer of flattened myoid cells (long thin arrow). The interstitial space (I) shows blood vessels (Bv) and Leydig cells (zigzag arrow), H&E X400. (**F**) The higher magnification of the figure (**B**) shows seminiferous tubule (ST) with different spermatogenic cells that include spermatogonia (thick arrow), primary spermatocytes (Ps), secondary spermatocytes (Sp), spermatids (S) and sperms (X). Sertoli cells (curved arrow) are seen on a regular basement membrane. The tubules are ensheathed by a single layer of flat myoid cells (long thin arrow). Clusters of Leydig cells (zigzag arrow) are seen in the narrow interstitial spaces (I), H&E X400. (**G**) The higher magnification of the figure (**C**) shows degenerated seminiferous tubule (ST*) with disorganization of germinal epithelium (G) and the presence of atrophic parts (*). Darkly stained nuclei (red short arrow) and multinucleated giant cells (green circle) are also observed. The interstitial space (I) showing inflammatory cells infiltration (white arrow) and vacuolated acidophilic hyaline material (V), H&E X400. (**H**) The higher magnification of the figure (**D**) shows one seminiferous tubule (ST*) lined by disorganized germinal epithelium (G) with atrophic parts in between (*). The other tubules appear nearly normal (ST) and retain their stratified germinal epithelium; spermatogonia (thick arrow), primary spermatocytes (Ps), secondary spermatocytes (Sp), spermatids (S) and sperms (X). Sertoli cells (curved arrow) are seen between spermatogenic cells. The tubule is ensheathed by a single layer of flattened myoid cells (long thin arrow). The interstitium contains clusters of Leydig cells (zigzag arrow) and some vacuolations are noticed (V). (**I**) Johnson’s testicular score. (**J**) The thickness of the capsule. (**K**) The thickness of the germinal epithelium. (**L**) The thickness of the interstitial space. (**M**) Number of Leydig cells. Scale bar = 50 μm, X400. Data are expressed as means ± SEM. *, *** indicate significant difference (*p* < 0.05).

The control and the HSP groups showed normal morphology. The majority of seminiferous tubules were rounded in shape with a regular contour and few interstitial spaces containing clusters of interstitial cells. The lumina of most tubules revealed aggregated sperm bundles in Figure 3A,B. A complete series of spermatogenic cells were seen (spermatogonia, primary spermatocytes, secondary spermatocytes and spermatids). The seminiferous tubules were covered by a single layer of myoid cells with flattened nuclei. The interstitial spaces were narrow and contained normal Leydig cells and blood vessels, as shown in Figure 3E,F.

Sections from CP-treated groups revealed degenerative testicular changes, including loss of their normal architecture, compared with the control group. Distorted seminiferous tubules with exfoliation of germ cells within their lumina and irregular outlines and germinal epithelium with some atrophic part. The spermatogenic cells appeared with darkly stained pyknotic nuclei and multinucleated giant cells were also seen. The interstitial tissues were wide and edematous. Numerous vacuolations were also seen, as shown in Figure 3C,G.

Sections from CP+HSP-treated groups showed a marked improvement in the histological structure of testicular tissue in comparison to the CP-treated group. The majority of the seminiferous tubules almost regained their normal architecture, had nearly regular outlines, and were lined by stratified germinal epithelium. A few seminiferous tubules were lined by disorganized germinal epithelium separated from the underlying basement membrane. The lumina of most of them showed aggregations of sperms, while others were empty. Normal blood vessels and clusters of Leydig cells were also noticed in the relatively narrow interstitium with some vacuolations still observed, as shown in Figure 3D,H.

Evaluation of Johnsen’s scores in histopathological sections indicated that there was a significant decrease in spermatogenesis quality in the CP-treated group compared to that in the control group (*p* < 0.001). When compared with the CP-treated group, the CP+HSP-treated group showed a significant rise, but was still significantly different to the control group in Figure 3I. Evaluation of the mean thickness of the capsule showed a significant increase in the CP-treated group in comparison to that of the control group (*p* < 0.001). The CP+HSP-treated group showed a significant decline when compared with the CP-treated group, but was still significantly different from that in the control group, as shown in Figure 3J. The CP-treated group’s mean thickness of the germinal epithelium was significantly decreased when compared to that of the control group (*p* < 0.001). The CP+HSP-treated group showed a significant increase in the thickness of germinal epithelium when compared with the CP-treated group, as shown in Figure 3K. The mean thickness of the interstitial space in the CP-treated group showed a significant increase in comparison to the control group (*p* < 0.001). When compared to the CP-treated group, the CP+HSP-treated group showed a significant decline in Figure 3L. The number of Leydig cells was significantly decreased in the CP-treated group compared to that in the control group (*p* < 0.001). However, the CP+HSP-treated group showed a significant increase in comparison to the CP-treated group, as shown in Figure 3M.

### 2.4. Effect on Testicular Immunohistochemistry of iNOS and P53

**Figure 4:**
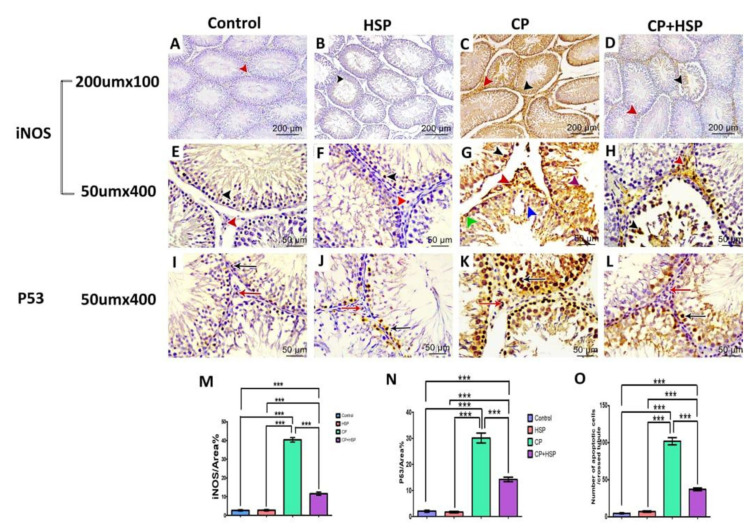
Effect of hesperidin administration in cyclophosphamide-induced testicular impairment in male rats on testicular immunohistochemical staining of iNOs and P53, as indicated by a positive immune reaction (arrowheads and arrows) in the cytoplasm and nuclei of the Leydig cells and germ cells of different studied groups (**A**–**O**). (**A**) Testicular immunohistochemical stained sections of iNOs in control-treated groups. Scale bar = 200 μm, X100. (**B**) Testicular immunohistochemical stained sections of iNOs in HSP-treated group. Scale bar = 200 μm, X100. (**C**) Testicular immunohistochemical stained sections of iNOs in CP-treated group. Scale bar = 200 μm, X100. (**D**) Testicular immunohistochemical stained sections of iNOs in CP+HSP-treated group. Scale bar = 200 μm, X100. (**E**) Testicular immunohistochemical stained sections of iNOs in control-treated groups. Scale bar = 50 μm, X400. (**F**) Testicular immunohistochemical stained sections of iNOs in HSP-treated group. Scale bar = 50 μm, X400. (**G**) Testicular immunohistochemical stained sections of iNOs in CP-treated group. Scale bar = 50 μm, X400. (**H**) Testicular immunohistochemical stained sections of iNOs in CP+HSP-treated group. Scale bar = 50 μm, X400. (**I**) Testicular immunohistochemical stained sections of P53 in control-treated groups. Scale bar = 50 μm, X400. (**J**) Testicular immunohistochemical stained sections of P53 in HSP-treated group. Scale bar = 50 μm, X400. (**K**) Testicular immunohistochemical stained sections of P53 in CP-treated group. Scale bar = 50 μm, X400. (**L**) Testicular immunohistochemical stained sections of P53 in CP+HSP-treated group. Scale bar = 50 μm, X400. (**M**) Immunostaining intensity of testicular iNOs (% area). (**N**) Immunostaining intensity of testicular P53 (% area). (**O**) Number of apoptotic cells per crossed tubule. Data are expressed as means ± SEM. *** indicate significant difference (*p* < 0.05).

Examination of the iNOS immunohistochemical stained section of the control and HSP-treated group revealed mild expression of iNOS immune reactivity in the cytoplasm of the germ cells of seminiferous tubules, shown in Figure 4A,B,E,F. The CP-treated group expressed a significant increase in iNOS immune reactivity in the cytoplasm of the germ cells and the interstitial cells, shown in Figure 4C,G, whereas administration of HSP led to a marked decrease in iNOS immunoreactivity in the cytoplasm of the germ cells of seminiferous tubules and the interstitial cells in Figure 4D,H. The mean area % of iNOS expression in the testes sections (X400) of the four experimental groups, was considerably higher in the CP-treated group in comparison to the control and CP+HSP-treated group, shown in Figure 4M.

In the seminiferous tubules of the control group and HSP-treated group, the P53 immunopositive reaction was primarily found in the nucleus of apoptotic cells, and is hardly noticeable in Figure 4I,J. Nevertheless, the seminiferous tubules of the CP-treated group had a large number of P53 immunopositive cells, shown in Figure 4K; however, the seminiferous tubules of the CP+HSP-treated group contained fewer P53 immunopositive cells, shown in Figure 4L. Regarding the average area % of P53 expression and the number of P53 immunopositive cells per tubule in the testes sections (X400) of the four experimental groups, the CP-treated group showed a significant increase compared to the control and CP+HSP-treated groups, shown in Figure 4N,O. The CP+HSP-treated group showed a significant decrease in comparison to the CP-treated group, shown in Figure 4N,O.

### 2.5. Effect on mRNA Expression of Hypothalamic KISS-1, KISS-1r, GnRH, Hypophyseal GnRHr and Testicular Steroidogenic Enzymes

**Figure 5:**
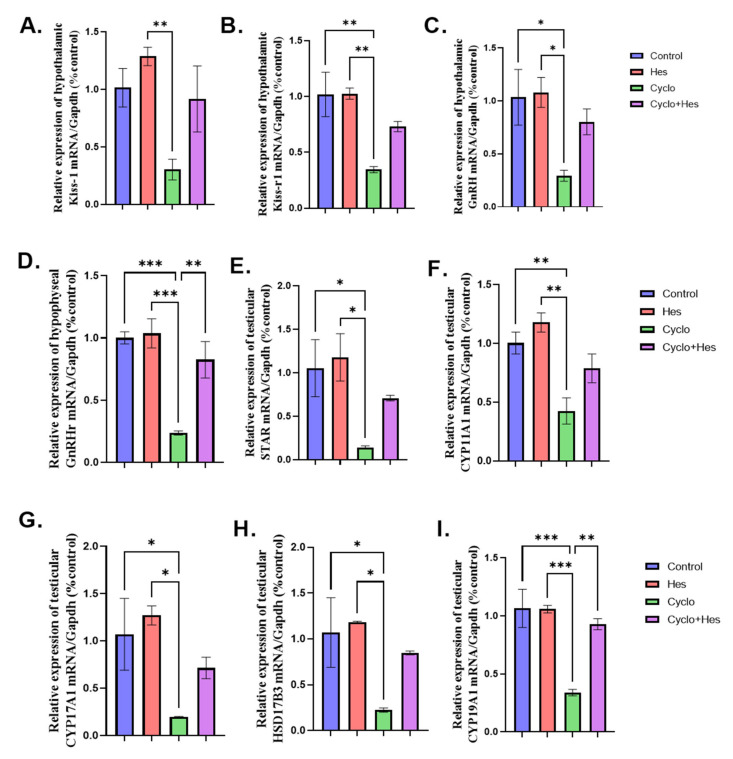
Effect of hesperidin administration in cyclophosphamide-induced testicular impairment in male rats on mRNA expression of hypothalamic KISS-1, KISS-1r, GnRH, hypophyseal GnRHr and testicular steroidogenic enzymes (**A**–**I**). (**A**) Hypothalamic KISS-1/Gapdh (% control), (**B**) mRNA expression of hypothalamic KISS-1r/Gapdh (% control), (**C**) mRNA expression of hypothalamic GnRH/Gapdh (% control), (**D**) mRNA expression of hypophyseal GnRHr/Gapdh (% control), (**E**) Testicular Star/Gapdh (% control), (**F**) Testicular Cyp11a1/Gapdh (% control), (**G**) Testicular Cyp17A1/Gapdh (% control), (**H**) Testicular HSD17B3/Gapdh (% control), and (**I**) Testicular Cyp19A1/Gapdh (% control). Data are expressed as means ± SEM. *, **, *** indicate significant difference (*p* < 0.05).

A significant downregulation in hypothalamic KISS-1, KISS-1r, and GnRH and hypophyseal GnRHr mRNA expression in the CP-treated group in comparison to that in the control group were noticed. Treatment with HSP significantly upregulated hypothalamic KISS-1 and GnRH and hypophyseal GnRHr, but not KISS-1r, in comparison to that in the CP-treated group, as shown in Figure 5A–D. In comparison to the control group, a significant downregulation in the testicular mRNA expression of StAR, CYP11A1, CYP17A1, HSD17B3, and CYP19A1 in the CP-treated group was noticed. In comparison to the CP-treated group, the CP+HSP-treated group showed considerable upregulation in the testicular StAR, CYP11A1, CYP17A1, HSD17B3, and CYP19A1, as shown in Figure 5E–I.

### 2.6. Effect on Testicular mRNA Expression of GLP-1, PGC-1, and PPAR-α

**Figure 6:**
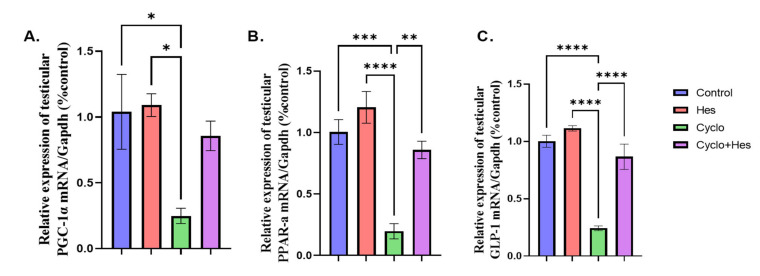
Effect of hesperidin administration in cyclophosphamide-induced testicular impairment in male rats on mRNA expression of testicular GLP-1, PGC-1, and PPAR-a (**A**–**C**). (**A**) Testicular PGC-1/Gapdh (% control), (**B**) mRNA expression of testicular PPAR-a/Gapdh (% control), and (**C**) mRNA expression of testicular GLP-1/Gapdh (% control). Data are expressed as means ± SEM. *, **, ***, **** indicate significant difference (*p* < 0.05).

A significant decrease in testicular GLP-1, PGC-1, and PPAR-a was shown in the CP-treated group compared to the control group in Figure 6A–C. HSP administration showed a significant increase in testicular GLP-1, PGC-1, and PPAR-α when compared to that in the CP-treated group, shown in Figure 6A–C.

### 2.7. Effect on Testicular Apoptotic and Inflammatory Marker

**Figure 7:**
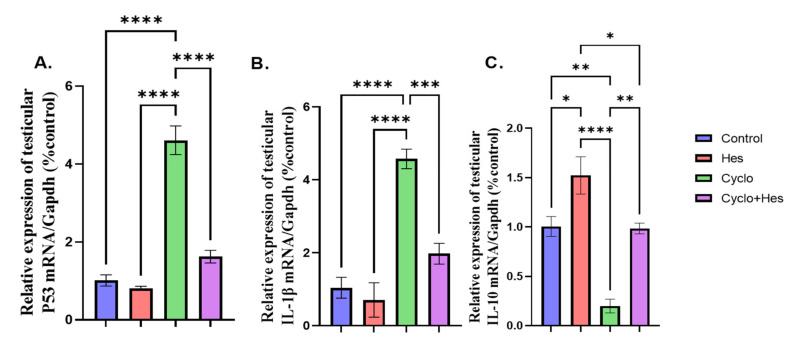
Effect of hesperidin administration in cyclophosphamide-induced testicular impairment in male rats on mRNA expression of testicular P53, IL1B, and IL10 (**A**–**C**). (**A**) Testicular P53/Gapdh (% control), (**B**) mRNA expression of testicular IL1B/Gapdh (% control), and (**C**) mRNA expression of testicular IL10/Gapdh (% control). Data are expressed as means ± SEM. *, **, ***, **** indicate significant difference (*p* < 0.05).

A significant upregulation in testicular P53 and IL1B mRNA expression and downregulation in testicular IL10 mRNA expression were detected in the CP-treated group when compared to that of the control group, as shown in Figure 7A–C. The CP+HSP-treated group showed significant downregulation of testicular P53 and IL1B and upregulation in testicular IL10 mRNA expression when compared to CP-treated group, shown in Figure 7A–C.

## 3. Discussion

Cancer is a leading cause of death worldwide [17]. As a result, researchers have been looking for different therapeutic strategies to improve the quality of life for patients. CP is a regularly used form of chemotherapy particularly in multiple myeloma, sarcoma, lymphoma, neuroblastoma, leukemia, and prostate and breast cancer [18]. CP has been shown to affect rapidly proliferating tissues such as gonads by interfering with their cell growth and differentiation [19]. CP treatment combined with several protective supplements, including polyphenols, vitamins, and minerals, has been suggested [20] to overcome such damaging impacts. HSP, owing to its antioxidant/anti-inflammatory effects, could be beneficial [21,22].

In the present work, the final body weight of rats receiving CP treatment was significantly decreased when compared with the control group, as previously reported [23]. A single intraperitoneal injection of a lesser dose of 120 mg/kg/b.wt [24] or 50 mg/kg/day for three days [25] also was reported to decrease body weight in rats. This decline could be caused by metabolic alterations causing appetite loss and decreased food intake. Yet, no weight change was noticed after administering CP in a single intraperitoneal dose of 200 mg/kg/b.wt. in rats [26]. HSP administration at a dose of 200 mg/kg/b.wt has been linked to improving the body weight in the aluminum phosphatide-treated group [27,28]. In the present study, the CP-treated group’s testicular weight significantly decreased when compared with the control group [29]. Administration of CP in a dose of 15 mg/kg once a week for 35 days has been linked to reduced testicular size in mice [30]. Other reports administering single CP at a dose of 100 mg/kg showed no difference in the weight of testis between groups [31,32]. HSP in a dose of 25–50 mg/kg for 60 days, revealed that HSP co-administration normalizes testicular weight [33]. Collectively, this could suggest a potential effect for the administration route, dose, treatment duration, and sensitivity of the animals in body and testicular weight changes.

The hypothalamic–pituitary–gonadal (HPG) axis controlling gonadal function in males starts by hypothalamic secretion of GnRH to stimulate the pituitary generation of FSH and LH. Additionally, FSH and LH play a role in regulating Leydig cell function to produce testosterone in males [8]. CP markedly reduced the serum levels of testosterone, FSH, LH, and prolactin [11], even at a single dose of 100 mg/kg [34]. The results of this study indicated that CP affected spermatogenesis by interfering with cellular processes as well as the pituitary–testicular axis. HSP significantly enhanced the serum testosterone, FSH, LH, and prolactin levels following bisphenol A administration [35,36]. Also, the administration of other flavonoids, including quercetin [11] or morin [37,38], or rutin [38], improved serum levels of testosterone, FSH, LH, and prolactin. This could be attributed to the protective roles of flavonoids in terms of anti-inflammatory and antioxidant ability.

CP disrupts the antioxidant defense mechanisms by producing large amounts of ROS in conjunction with its harmful metabolite, acrolein, which is known to increase oxidative stress, causing a marked reduction in GPx and TAC levels and a marked elevation in MDA levels [39,40]. Oxidative stress is a major contributor to male infertility [41] via modifications in microvascular blood flow leading to elevated rates of germ cell death. HSP co-administration reduced the level of the lipid peroxidation marker, MDA, and also strengthened the body’s natural antioxidant defense system by boosting the activity of antioxidant enzymes such as SOD and GPx [27,42,43,44]. Oxidative stress, lipid peroxidation, and inflammation usually manifest histological and functional abnormalities in testicular tissue [45,46]. CP intraperitoneal administration at a dose of 5 mg/kg daily for four weeks, showed intraepithelial vacuoles as an indication of seminiferous tubules atrophy, as well as spacing and separation of germ cells, which may be caused by sloughing and exfoliation of the germ cells due to the primary impact on the cell-to-cell junction in between Sertoli cells and germ cells [47,48].

Other reports of single CP administration at a dose of 200 mg/kg showed marked testicular damage, including interstitial bleeding, and separation of spermatogenic cells with the presence of vacuoles, and explained how CP could cause oxidative stress, lipid peroxidation, and apoptosis [49]. CP intraperitoneal administration at a dose of 60 mg/kg per week for eight weeks revealed that the seminiferous tubules exhibited morphological changes, including interstitial edema, vacuolization, multinucleated giant cell development, desquamation, degeneration, and disorganization. In addition, reactive oxygen species and oxidative stress have been linked to the pathogenesis of CP toxicity [50].

The CP-treated group revealed a significant increase in iNOS, only formed during inflammation, ischemia, and apoptosis [51], immune reactivity in the cytoplasm of the cells of the seminiferous tubules [52]. Exposure to cisplatin, aluminum chloride, methotrexate, and silica nanoparticles reported an increase in testicular iNOS expression [53,54,55,56]. Anticancer drugs, including cyclophosphamide, methotrexate, tamoxifen, doxorubicin, and 5-fluorouracil administration, have been associated with the induction of oxidative stress and inflammation [57]. Such results, confirmed by another flavonoid (quercetin), [54] found that coadministration of quercetin to the aluminum chloride-treated group decreased the iNOS expression in testis tissue. HSP caused a marked decrease in the CP-induced expression of iNOS in the liver [58] and renal tissue [59]. Coadministration of another antioxidant as melatonin decreased the expression of iNOS in testicular tissue in the cisplatin-treated group [60].

Increased testicular apoptosis, as indicated by increased P53 expression, results in spermatogenic arrest and a reduction in spermatogonia, spermatocytes, and spermatid numbers [61]. The CP-treated group revealed a marked increase in P53 immune reactivity in the nucleus of the cells of the seminiferous tubules and revealed an increased number of apoptotic cells in the CP-treated group [62]. Exposure to potassium dichromate, D-gal/NaNO_2_, bleomycin, etoposide, and cisplatin resulted in an increase in testicular P53 expression [63,64,65]. Treatment with hesperidin might protect mice’s testis against apoptosis [66]. HSP administration at a dose of 50 mg/kg daily via gavage for 14 days, reported mitigated testicular alterations brought on by cisplatin [67]. Additionally, HSP treatment reduced the testicular seminiferous tubules deterioration process and lowered ischemia/reperfusion-induced reproductive damage [68]. HSP caused a marked decrease in the expression of P53 in the corneal tissue [69], liver [22], and colon [70]. Regarding other flavonoids, co-administration of quercetin to the cisplatin-treated group decreased the expression of P53 in testicular tissue [71].

The mean testicular Johnsen’s score in the CP-treated group decreased significantly in comparison with the control group [52]. The thickness of germinal epithelium had significantly decreased and the lumina were large and empty [72]. They linked these findings to the inhibition of B-spermatogonia mitosis, which denotes an extension of the G1 phase of the cell cycle growth. Additionally, it has been demonstrated that this epithelial thinning results in a deficiency in sperm production. A significant decline in epithelial height and increase in the interstitial space in the cisplatin-treated group [73] could be attributed to oxidative stress that was responsible for all cisplatin-induced damage in the testis. The thickness of the capsule of the testis was significantly increased in the iprodione-treated group [35]. The number of Leydig cells decreased significantly in the CP-treated group [74,75]. In the present study, there was a significant improvement in testicular Johnsen’s score in the CP+HSP-treated group when compared to the CP-treated group. This result was consistent with previous reports indicating that diabetic rats treated with HSP showed considerable improvement in the mean testicular biopsy score [76].

In the present study, the number of Leydig cells in the CP+HSP-treated group increased significantly when compared to the CP-treated group [77]. HSP treatment with cisplatin causes considerable improvement in the thickness of germinal epithelium [67]. Quercetin co-treatment showed a significant reduction in the interstitial space in the arsenic-treated group [78]. Melatonin co-treatment showed a significant reduction in the thickness of tunica albuginea in the taxol-treated group [79].

In this study, there was significant downregulation of hypothalamic KISS, KISSr, GnRH, and hypophyseal GnRHr in the CP-treated group when compared with the control group. Oxidative stress induced by extensive exercise has been reported to downregulate the expression of KISS, KISSr, GnRH, and GnRHr [80]. A significant upregulation in gene expression of hypothalamic KISS, GnRH, and hypophyseal GnRHr in the CP+HSP-treated group if compared with the CP-treated group was noticed. This could be attributed to the flavonoid’s ability to prevent tissue damage, prevent the inactivation of steroidogenesis, and increase gonadotropin release [81,82]. CP treatment significantly decreases steroidogenic genes that include StAR, CYP11A1, CYP17A1, HSD17B, and CYP1719A1 compared with other groups [11,83]. Cisplatin has been reported to induce similar impacts [84,85]. HSP administration enhances testicular functions through the upregulation of steroidogenesis-related genes [86]. Additionally, isorhamnetin, a bioflavonoid treatment, significantly improved testosterone production through the upregulation of steroidogenic genes and antioxidant ability [87]. On the other hand, studies have also suggested that flavonoids may interact with estrogen receptors (ERs) to modulate the activity of the endocrine system [88,89]. This interaction has been linked to a variety of health outcomes, including testicular dysfunction in rats [88]. The interaction between flavonoids and ERs is complex and depends on the type of flavonoid, duration of administration and its concentration. Such interaction should be in focus in future research.

GLP-1 is essential for maintaining male fertility, since it controls spermatogenesis and steroidogenesis directly. It is released by Leydig cells and works on Sertoli, germinal epithelial, and Leydig cells (all of which have GLP-1 receptors). This procedure increases the metabolism of Sertoli cells and raises sperm cell quality [90]. PPAR-α and PGC-1α, stimulate fatty acid oxidation and impact the testicular energy balance [91]. Testicular torsion, similar to CP administration, caused significant downregulation of testicular GLP-1, PPAR-α, and PGC-1α and affect the metabolism of testicular cells and worsens oxidative stress by raising reactive oxygen species and reactive nitrogen species, which promote apoptosis and inflammation inside testis [92]. A significant upregulation of testicular GLP-1, PPAR-α, and PGC-1α in the CP+HSP-treated group was noticed, indicating a positive effect on testicular metabolism. CP-treated rats showed a significant upregulation of testicular apoptotic P53 [83] and pro-inflammatory IL1β and downregulation of anti-inflammatory IL10. Cisplatin caused a significant elevation in pro-inflammatory IL1β and significant downregulation of the anti-inflammatory IL10 gene respectively [84,93]. HSP’s antioxidant properties cleared the ROS, preventing the pro-inflammatory genes from activating, and protecting testicular tissue from inflammation [94]. CP and HSP-combined treatment downregulated the testicular apoptotic P53 gene and pro-inflammatory IL1β and significant upregulated the anti-inflammatory IL10 gene when compared with CP-treated group. Co-treatment with bilobetin, a natural bioflavonoid, significantly reduced testicular P53 expression and significantly increased testicular IL10 expression in the cisplatin-treated group [95].

## 4. Materials and Methods

### 4.1. Chemicals

Cyclophosphamide in the form of powder acquired from Baxter oncology GmbH in Germany. CP was injected at a dose of 150 mg/kg/B.wt. Hesperidin (>80% purity powder CAS NO 520-26-3) was produced by Sigma-Aldrich Company St. Louis, MO, USA, and acquired from Sigma-Egypt. Hesperidin was orally administrated by gavage at a dose of 200 mg/kg/B.wt.

### 4.2. Experimental Animals

Thirty-two adult healthy male Sprague Dawley rats (12–14 weeks) weighing 210 ± 10 g were obtained from Zagazig scientific and medical research center (ZSMRC). The animals were then kept at a constant 23 ± 2 °C and operated on a 12-h light/12-h dark cycle. Throughout the study, the animals were kept on a regular diet and ad libitum water supply. All rats received humane care and the experimental methods were approved by the Institutional Animal Care and Use Committee of Zagazig University (No. ZU-IACUC/3/F/172/2019).

### 4.3. Experimental Design and Sample Collection

The rats were randomly assigned into four main groups (n = 8), namely, the control group that received normal saline for eight days, the HSP-treated group that received HSP 200 mg/kg/d orally for eight days [27,96], the CP-treated group that received CP 150 mg/kg single intraperitoneal injection on the 1st day of the experiment [87], and CP+HSP-treated group that received CP 150 mg/kg single intraperitoneal injection on the 1st day of the experiment and HSP 200 mg/kg/d orally for eight days. Rats were euthanized 48 h after the last HSP dose. The body weights were determined and venous blood samples were taken from their retro-orbital plexus using a capillary glass tube, blood was left to clot at room temperature, then centrifuged at 3000 rpm for 10 min to separate the serum. The samples were then kept at −20 °C to be used subsequently for hormonal assay estimation. Then, the rats were anesthetized by intraperitoneal injection of thiopental (75 mg/kg BW) and subjected to cervical dislocation, the abdomen was then opened to collect both testes outside the body, weighted and then divided into three parts; the first part was collected on 10% neutral buffered formalin for histopathological and immunohistochemical examination, the second part (30 mg) was removed directly on liquid nitrogen and then kept at −80 °C to be used for total RNA extraction and the third part (1 g) was homogenized to be used for different biochemical tests. After being dissected, the hypothalamus and pituitary gland were stored in liquid nitrogen and kept there at −80 °C until total RNA extraction as previously described [97].

### 4.4. Hormonal and Biochemical Analysis

The levels of serum of FSH, LH, total testosterone, and prolactin were determined by using a commercially available rat enzyme-linked immunosorbent assay (ELISA) (Catalog No. CSB-E12654r) for LH as well as (Catalog No. CSB-E06869r) for FSH and (Catalog No. MBS282195) for testosterone and (Catalog No. CSB-E06881r) for prolactin [98,99]. The levels of malondialdehyde (MDA), glutathione peroxidase enzyme activity (GPx), and total antioxidant capacity (TAC) were assessed using (Catalog No. (ELA-E0597r) for MDA, (Catalog No. ELA-E0295r) for GPx, (Catalog No. STA-360) for TAC.

### 4.5. Real-Time Quantitative RT-PCR (qRT-PCR) Analysis

Briefly, total RNA was extracted using Trizol (Invitrogen; Thermo Fisher Scientific, Waltham, MA, USA), and for evaluating the RNA quality, the A260/A280 ratio was analyzed using the NanoDrop VR ND-1000 Spectrophotometer (NanoDrop Technologies; Wilmington, DE, USA). For cDNA synthesis, a High-Capacity cDNA Reverse Transcription Kit cDNA Kit (Applied Biosystems™, USA) was used, followed by the preparation of the primers according to their manufacturer instructions, Sangon Biotech (Beijing, China), as provided in Table 1.

Real-time RT-PCR was performed in Mx3005P real-time PCR system (Agilent Stratagene, USA) using TOPrealTM qPCR 2X PreMIX (SYBR Green with low ROX) (Cat. # P725 or P750) (Enzynomics, Korea) following the manufacturer’s instructions. The PCR cycling conditions included initial denaturation at 95 °C for 12 min followed by 40 cycles of denaturation at 95 °C for 20 s, annealing at 60 °C for 30 s, and extension at 72 °C for 30 s. A melting curve analysis was performed following PCR amplification. The expression level of the target genes was normalized using the mRNA expression of a known housekeeping gene, Gapdh. Results are expressed as fold-changes compared to the control group following the 2^−ΔΔCt^ method [100].

### 4.6. Histopathological and Immunohistochemical Examination

All the testicular specimens from all groups were installed in Bouin solution for 4 to 5 h till converted to a hard consistency then kept for paraffin block preparation. For the light microscopic analysis, tissue sections of 5 μm thickness were stained with H and E stain to examine the structural light microscopic alterations [101]. For immunohistochemical staining, a rabbit monoclonal antibody of IgG type was designed for specific localization of inducible nitric oxide synthase (iNOS) marker for oxidative stress and P53 (apoptosis marker) in paraffin sections. The kits were delivered from DAKO life trade Egypt (Catalog No. A0312 for iNOS and Catalog No. A5761 for P53). Following the manufacturer’s recommendations, sections were inspected and photographed by light microscope LEICA DM500 in the Anatomy Department, Faculty of Medicine, Zagazig University

The Johnsen scoring system was used to assess the histological alterations in testicular tissue. Each tubule received a Johnsen’s score between 1 (extremely poor) and 10 (excellent) following Johnsen’s criteria [102]. Image J analysis software (Fiji image j; 1.51 n, NIH, USA) was used for measuring the thickness of the capsule, the thickness of the germinal epithelium, the thickness of the interstitial space, and the count of Leydig cells in sections stained with H&E per 100 high powers fields for the thickness of capsule and 400 high powers fields for the thickness of the germinal epithelium, the thickness of the interstitial space and count of Leydig cells [103]. After immunostaining, the number of apoptotic cells (in P53 stained sections) was counted and analysis of the mean area % of P53 (in P53 stained sections) or iNOS (in iNOS stained sections) was performed in nearly 150 seminiferous tubules from 8 animals/group, and the findings were statistically analyzed [104].

### 4.7. Statistical Analysis

Continuous variables were represented by the mean ± standard error mean (SEM). All data were normally distributed and were analyzed by one-way analysis of variance (ANOVA) followed by Tukey’s honest significant difference test in homogenous data for multiple group comparison. *p* values less than 0.05 (*p* < 0.05) was statistically significant using the statistical software package SPSS for Windows (Version 20; SPSS Inc., Chicago, IL, USA).

## 5. Conclusions

Our study concludes that hesperidin’s antioxidant, anti-inflammatory, and anti-apoptotic activities could modulate testicular disturbances induced by cyclophosphamide. The findings of our study raise the prospect of hesperidin, a bioflavonoid, as a therapeutic intervention for delaying testicular dysfunction. Such findings could be an important entry point for preserving fertility while using antineoplastic drugs such as cyclophosphamide against a wide spectrum of malignancies. Although the therapeutic applications of bioflavonoids, including hesperidin, should be promising, any future clinical application should be preceded by rigorous clinical studies.

## Figures and Tables

**Table 1:** Forward and reverse primers sequence of targeted genes.

Gene	Forward Primer Sequence (5′ to 3′)	Reverse Primer Sequence (5′ to 3′)	Product Size	Accession No.
GLP1	CACCTCCTCTCAGCTCAGTC	CGTTCTCCTCCGTGTCTTGA	128	NM_012707.2
Pparα	GTCCTCTGGTTGTCCCCTTG	GTCAGTTCACAGGGAAGGCA	176	NM_013196.2
PGC1α	TTCAGGAGCTGGATGGCTTG	GGGCAGCACACTCTATGTCA	70	NM_031347.1
*Gapdh*	GCATCTTCTTGTGCAGTGCC	GGTAACCAGGCGTCCGATAC	91	NM_017008.4
Kiss-1	TGCTGCTTCTCCTCTGTGTGG	ATTAACGAGTTCCTGGGGTCC	110	NM_181692.1
Kiss-1r	CTTTCCTTCTGTGCTGCGTA	CCTGCTGGATGTAGTTGACG	102	NM_023992.1
GnRH1	AGGAGCTCTGGAACGTCTGAT	AGCGTCAATGTCACACTCGG	100	NM_012767.2
GnRHr	TCAGGACCCACGCAAACTAC	CTGGCTCTGACACCCTGTTT	182	NM_031038.3
StAr	CCCAAATGTCAAGGAAATCA	AGGCATCTCCCCAAAGTG	187	NM_031558.3
CYP11A1	AAGTATCCGTGATGTGGG	TCATACAGTGTCGCCTTTTCT	127	NM_017286.3
CYP17A1	TGGCTTTCCTGGTGCACAATC	TGAAAGTTGGTGTTCGGCTGAAG	90	NM_012753.2
HSD17B3	AGTGTGTGAGGTTCTCCCGGTACCT	TACAACATTGAGTCCATGTCTGGCCAG	161	NM_054007.1
CYP19A1	GCTGAGAGACGTGGAGACCTG	CTCTGTCACCAACAACAGTGTGG	178	NM_017085.2
IL10	GTAGAAGTGATGCCCCAGGC	AGAAATCGATGACAGCGTCG	116	NM_012854.2
IL1β	CACCTCTCAAGCAGAGCACAGA	ACGGGTTCCATGGTGAAGTC	81	NM_031512.2
P53	GTTCGTGTTTGTGCCTGTCC	TGCTCTCTTTGCACTCCCTG	108	NM_030989.3

## Data Availability

The data presented in this study are available upon reasonable request from the corresponding author. The data are not publicly available as it contains information that could compromise the privacy of an ongoing research.
